# Diagnostic performance of a multiplexed gastrointestinal PCR panel for identifying diarrheal pathogens in children undergoing hematopoietic stem cell transplant

**DOI:** 10.1007/s12519-023-00776-w

**Published:** 2024-02-17

**Authors:** Yue Tao, Cheng-Juan Luo, Bing-Hua Zhang, Xin-Yan Shen, Rui-Ke Zhao, Bei-Ying Ma, Nan Shen, Chang-Ying Luo, Jian-Min Wang, Yi-Jun Xia, Li Xie, Jing Chen, Xi Mo

**Affiliations:** 1 Pediatric Translational Medicine Institute, Shanghai Children’s Medical Center, School of Medicine, Shanghai Jiao Tong University, 1678 Dongfang Rd., Shanghai, 200127 China; 2 Department of Hematology and Oncology, Shanghai Children’s Medical Center, School of Medicine, Shanghai Jiao Tong University, 1678 Dongfang Rd., Shanghai, 200127 China; 3 Department of Infectious Diseases, Shanghai Children’s Medical Center, School of Medicine, Shanghai Jiao Tong University, Shanghai, China; 4 Medical Affairs, BioMérieux (Shanghai) Company, Limited, Shanghai, China; 5 Clinical Research Institute, School of Medicine, Shanghai Jiao Tong University, Shanghai, China

**Keywords:** Children, Diarrhea, Hematopoietic stem cell transplantation, Multiplexed polymerase chain reaction

## Abstract

**Background:**

Diarrhea is a common complication of hematopoietic stem cell transplantation (HSCT) and is associated with substantial morbidity, but its etiology is often unknown. Etiologies of diarrhea in this population include infectious causes, chemotherapy- or medication-induced mucosal injury and graft-versus-host disease (GVHD). Distinguishing these potential causes of diarrhea is challenging since diarrheal symptoms are often multifactorial, and the etiologies often overlap in transplant patients. The objectives of this study were to evaluate whether the FilmArray gastrointestinal (GI) panel would increase diagnostic yield and the degree to which pre-transplantation colonization predicts post-transplantation infection.

**Methods:**

From November 2019 to February 2021, a total of 158 patients undergoing HSCT were prospectively included in the study. Stool specimens were obtained from all HSCT recipients prior to conditioning therapy, 28 ± 7 days after transplantation and at any new episode of diarrhea. All stool samples were tested by the FilmArray GI panel and other clinical microbiological assays.

**Results:**

The primary cause of post-transplantation diarrhea was infection (57/84, 67.86%), followed by medication (38/84, 45.24%) and GVHD (21/84, 25.00%). Ninety-five of 158 patients were colonized with at least one gastrointestinal pathogen before conditioning therapy, and the incidence of infectious diarrhea was significantly higher in colonized patients (47/95, 49.47%) than in non-colonized patients (10/63, 15.87%) (*P* < 0.001). Fourteen of 19 (73.68%) patients who were initially colonized with norovirus pre-transplantation developed a post-transplantation norovirus infection. Twenty-four of 62 (38.71%) patients colonized with *Clostridium difficile* developed a diarrheal infection. In addition, FilmArray GI panel testing improved the diagnostic yield by almost twofold in our study (55/92, 59.78% vs. 30/92, 32.61%).

**Conclusions:**

Our data show that more than half of pediatric patients who were admitted for HSCT were colonized with various gastrointestinal pathogens, and more than one-third of these pathogens were associated with post-transplantation diarrhea. In addition, the FilmArray GI panel can increase the detection rate of diarrheal pathogens in pediatric HSCT patients, but the panel needs to be optimized for pathogen species, and further studies assessing its clinical impact and cost-effectiveness in this specific patient population are also needed.

**Graphical abstract:**

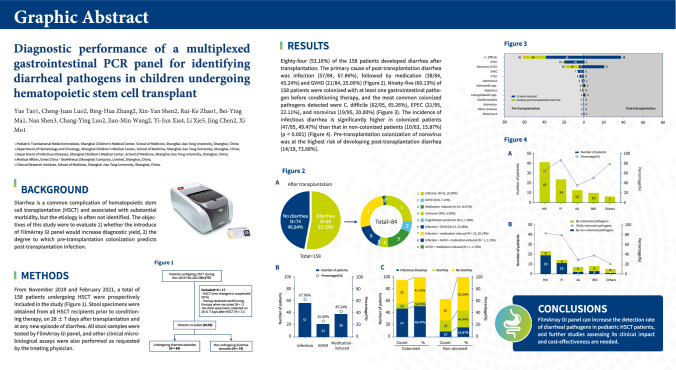

## Introduction

Diarrhea is a common complication of hematopoietic stem cell transplantation (HSCT), occurring in 43% to 91% of patients, and is associated with substantial morbidity [1–3]. Etiologies of diarrhea in this population include infectious causes, chemotherapy- or medication-induced mucosal injury and graft-versus-host disease (GVHD). Distinguishing these potential causes of diarrhea is challenging since diarrheal symptoms are often multifactorial, and the etiologies often overlap in transplant patients [4, 5]. Positive results from microbiologic tests in stool samples may improve antimicrobial stewardship, improve infection prevention, and decrease the use of other diagnostic modalities, although they do not rule out other clinically important causes of gastrointestinal (GI) symptoms. However, conventional stool testing methods (e.g., stool culture, serology tests, ova and parasite examination) are unable to detect most diarrheagenic *Escherichia coli* (*E*. *coli*) and have limited sensitivity for detecting other pathogens [6, 7]. Compared to conventional stool testing, multiplexed polymerase chain reaction (PCR) panels have shown increased sensitivity for detecting GI pathogens in the general population [8, 9], but studies focused on transplant patients are scarce and limited to adults [10]. The clinical diagnostic application of multiplexed PCR panels in children undergoing HSCT remains to be evaluated.

Asymptomatic colonization with GI pathogens is well reported in the literature, and most studies have focused on asymptomatic colonization with *Clostridium difficile* (*C. difficile*) [11–13]. For vulnerable populations, such as patients undergoing HSCT, GI carriage of microbial pathogens may be a reservoir from which pre-transplantation colonization may develop to post-transplantation infection. Determining the predictive value of GI pathogen colonization before HSCT on the development of diarrhea with certain infectious pathogens post-transplantation can provide a positive effect on infection control. However, the significance of certain studies has been underestimated. The only study from Kubiak et al*.* reported that 37% of adult patients were colonized with at least one pathogen before transplantation, and 48.9% of those colonized patients developed a post-transplantation diarrhoeal infection [14]. In pediatric HSCT patients, there are no related data yet.

In this study, we used a BioFire FilmArray GI panel (bioMérieux, Marcy l’Etoile, France) to evaluate whether the introduction of the FilmArray GI panel would increase diagnostic yield and improve the understanding of the epidemiology of diarrhea and the degree to which pre-transplantation colonization predicts post-transplantation infection in pediatric HSCT patients.

## Methods

### Study population

**Fig. 1:**
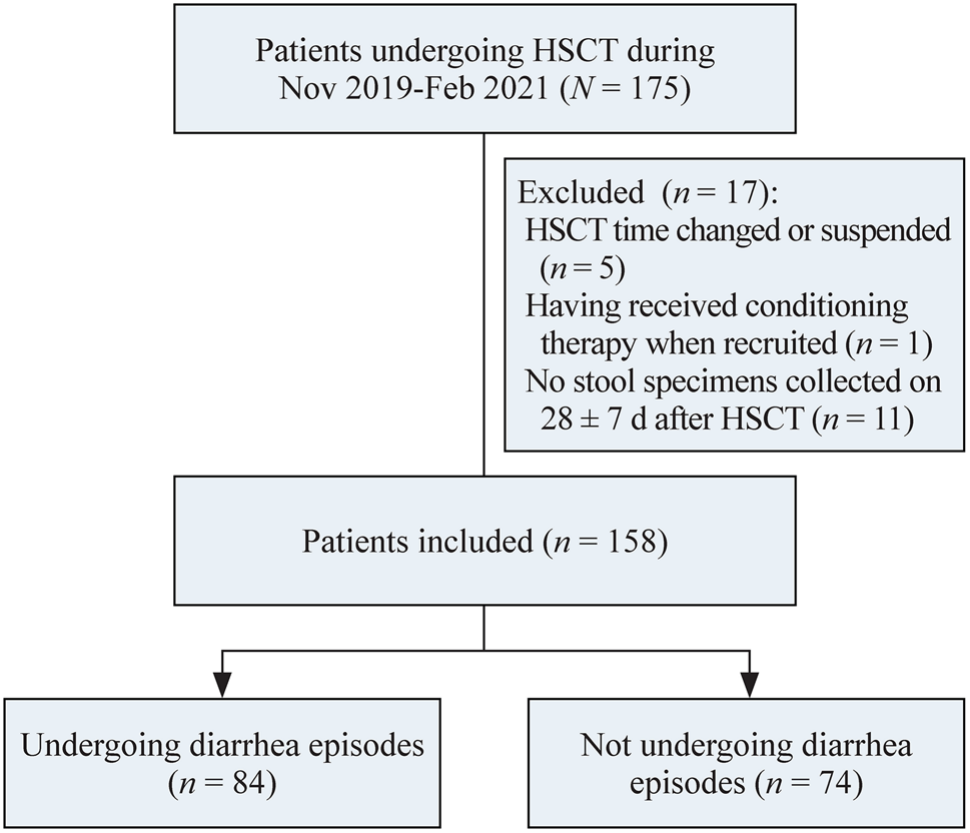
Flowchart of patient recruitment. A total of 158 patients were recruited from 175 patients undergoing HSCT during the study period. Eighty-four of the 158 patients experienced diarrhea episodes after transplantation, while the other 74 patients did not. *HSCT* hematopoietic stem cell transplantation

This prospective study of patients undergoing either allogeneic or autologous HSCT was performed at Shanghai Children’s Medical Center between November 2019 and February 2021. A total of 158 patients were included in the study, according to our inclusion/exclusion criteria (Fig. 1). Patient demographics, underlying diseases, and transplant types were obtained from the medical records. The study was approved by the Institutional Review Board and the Ethics Committee of Shanghai Children’s Medical Center (SCMCIRB-K2018109), and written informed consent was obtained from each patient and/or their parents.

### Study design

Stool specimens were obtained from all HSCT recipients prior to conditioning therapy and 28 ± 7 days after transplantation. In addition, stool specimens were also obtained at any new episode of diarrhea. All these specimens were tested with the FilmArray GI panel within 24 hours after collection. Post-transplantation diarrhea was defined as ≥ 3 unformed stools per day [15]. Infectious diarrhea was defined as the detection of the pathogen or toxin in diarrhea episodes during transplantation admission by several methods indicated below. The diagnosis of noninfectious causes of diarrhea, including GVHD and medication-induced diarrhea, as well as infection-causing pathogens were determined by retrospective, in-depth chart review performed independently by two physicians with expertise in HSCT, according to established criteria [16].

### FilmArray GI panel testing

The FilmArray GI panel targets 22 pathogens, including 13 bacterial pathogens: *Campylobacter* spp*.* (*jejuni*, *coli*, and *upsaliensis*), *Plesiomonas shigelloides*, *Salmonella* spp., *Yersinia enterocolitica*, *Vibrio* spp*.* (*parahaemolyticus*, *vulnificus*, and *cholerae*), enteroaggregative *E. coli* (EAEC), enteropathogenic *E. coli* (EPEC), enterotoxigenic *E. coli* (ETEC), Shiga toxin-producing *E. coli*, *Shigella*/enteroinvasive *E. coli*, and *C. difficile*; five viral pathogens: adenovirus F40/41, astrovirus, norovirus GI/GII, rotavirus A, and sapovirus (I, II, IV, and V); and four protozoan pathogens: *Cryptosporidium*, *Cyclospora cayetanensis*, *Entamoeba histolytica*, and *Giardia lamblia*. The FilmArray GI assay was performed according to the manufacturer’s instructions. Comprehensive results were available within approximately 1 hour after a 2-minute operating time.

### Microbiological evaluation

In diarrhea episodes, other clinical microbiological assays were also performed as requested by the treating physician. Routine microbiological assays included stool culture (for the presence of pathogenic bacteria), stool microscopic examination (for the presence of parasites), stool antigen tests (for rotavirus, adenovirus, and norovirus), stool real-time quantitative PCR (RT‒qPCR) test [for cytomegalovirus (CMV), Epstein–Barr virus (EBV), BK virus, human herpesvirus 6 (HHV6), adenovirus, norovirus, and human parvovirus B19], and metagenomic next-generation sequencing (mNGS) for intestinal mucosa specimens.

### Statistical analyses

For each patient who developed diarrhea after transplantation, we first analyzed the proportion of diarrhea due to different causes, including GVHD-, infection-, and medication-induced diarrhea. We then determined the proportions of patients who tested positive with the FilmArray GI panel prior to conditioning therapy and 28 ± 7 days after transplantation. Next, we assessed the proportions of patients who developed diarrheal infections due to their pre-transplantation colonizing pathogens detected by the FilmArray GI panel. The proportions of patients with different underlying diseases and who developed diarrheal infection due to colonizing pathogens were compared using the Chi-square test when the expected number of events was five or more or using Fisher’s exact test when the expected number was less than five. Statistical analyses and figures were performed with GraphPad Prism, version 8.3.0 (GraphPad Software). A two-sided *P* < 0.05 was considered statistically significant.

## Results

### Clinical characteristics of patients

A total of 158 patients undergoing HSCT, aged from 9 months to 16 years, were enrolled in the present study between November 2019 and February 2021. The median patient age was 6.00 years, with 37.97% of patients being female and 96.84% being allogeneic HSCT recipients. The most common underlying diseases were hematological malignancies (40.51%), aplastic anemia (23.42%), primary immunodeficiency (17.72%), and inherited metabolic disorders (12.66%).

Ninety-five of the 158 (60.13%) patients tested positive for at least one GI pathogen before transplantation and were therefore classified as colonized patients. There were no significant differences regarding age, stem cell source or outcomes in colonized or non-colonized patients (Table 1).

**Table 1:** Clinical characteristics of patients colonized or not colonized with a gastrointestinal pathogen before HSCT

Variables	Colonized (*n* = 95)	Non-colonized (*n* = 63)	Total (*N* = 158)	*P* value
Age (y)				0.150^a^
*n*	95	63	158	
Mean (SD)	5.61 (4.31)	6.59 (3.95)	6.00 (4.18)	
Sex, *n* (%)				0.042^b^
Male	65 (68.42)	33 (52.38)	98 (62.03)	
Female	30 (31.58)	30 (47.62)	60 (37.97)	
Underlying malignancy, *n* (%)				0.002^b^
Hematological malignancies	41 (43.16)	23 (36.51)	64 (40.51)	
Primary immunodeficiency	24 (25.26)	4 (6.35)	28 (17.72)	
Aplastic anemia	13 (13.68)	24 (38.10)	37 (23.42)	
Inherited metabolic disorders	10 (10.53)	10 (15.87)	20 (12.66)	
Solid tumors	4 (4.21)	1 (1.59)	5 (3.16)	
Inherited bone marrow failure	2 (2.11)	0 (0.00)	2 (1.27)	
Others^c^	1 (1.05)	1 (1.59)	2 (1.27)	
Stem cell source, *n* (%)				0.356^b^
Allogeneic	91 (95.79)	62 (98.41)	153 (96.84)	
Autologous	4 (4.21)	1 (1.59)	5 (3.16)	
Outcome, *n* (%)				0.148^b^
Disease free survival	81 (85.26)	46 (73.02)	127 (80.38)	
Survival with disease	2 (2.11)	5 (7.94)	7 (4.43)	
Cause-specific death	4 (4.21)	6 (9.52)	10 (6.33)	
Transplantation related death	8 (8.42)	6 (9.52)	14 (8.86)	
Diarrhea after HSCT, *n* (%)				0.074^b^
Yes	56 (58.95)	28 (44.44)	84 (53.16)	
No	39 (41.05)	35 (55.56)	74 46.84)	

*HSCT* hematopoietic stem cell transplantation, *SD* standard deviation. ^a^Linear model ANOVA; ^b^Pearson’s Chi-squared test; ^c^others include one with thalassemia major (colonized) and one with systemic Epstein–Barr virus-positive T-cell lymphoproliferative disease (non-colonized)

### Etiologies of diarrhea after transplantation

**Fig. 2:**
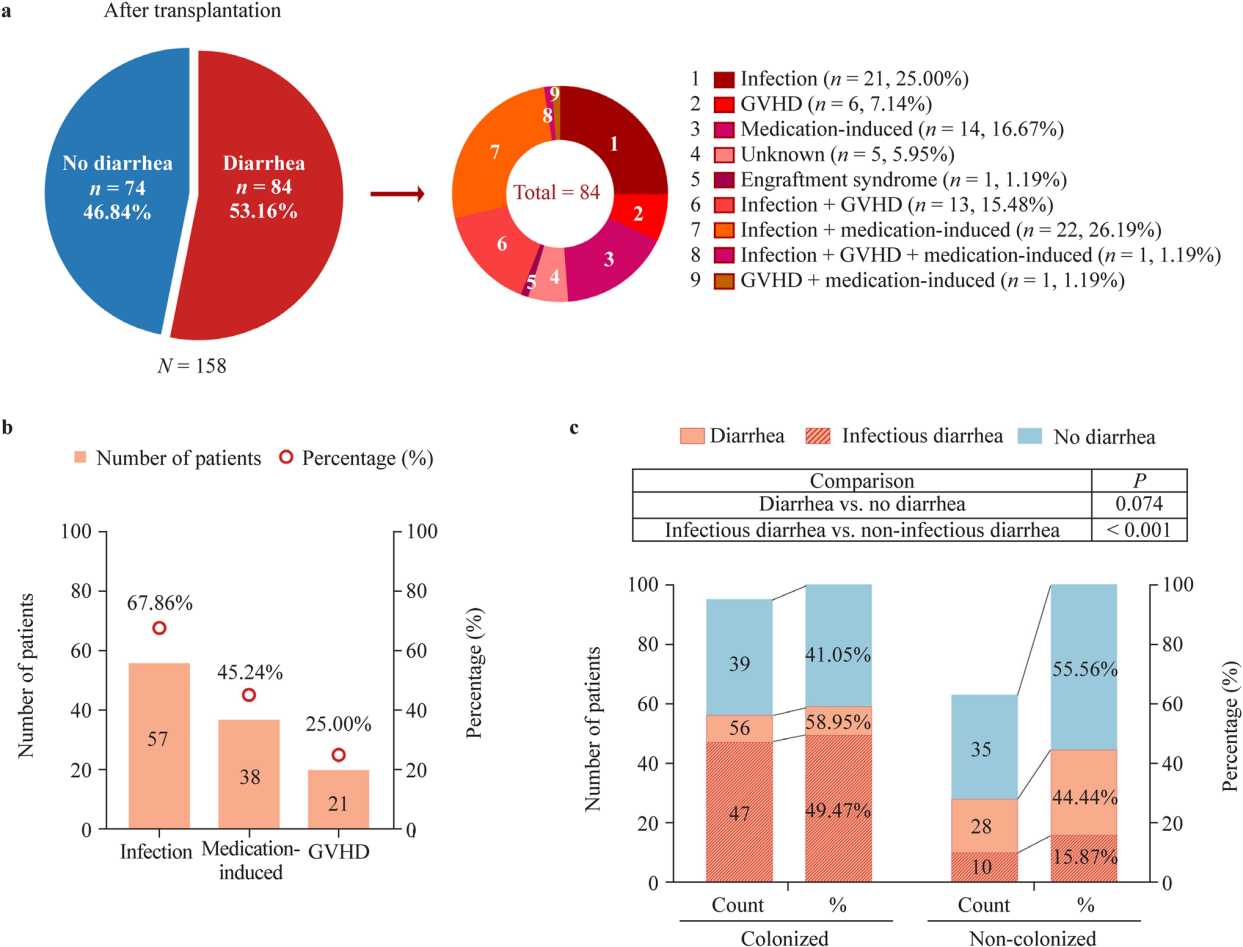
Etiologies of diarrhea after transplantation. **a** Etiologies of diarrhea in 84 patients who experienced diarrhea episodes after transplantation; **b** etiologies of diarrhea that combined all factors, including infection, GVHD and medication; **c** the incidence of diarrhea and infectious diarrhea in colonized and non-colonized patients. *GVHD* graft-versus-host disease

Eighty-four of the 158 (53.16%) patients developed diarrhea after transplantation (Fig. 1). In half of these patients, diarrhea was considered to be caused by a single factor, including infection (21/84, 25.00%), medication-induced diarrhea (14/84, 16.67%), GVHD (6/84, 7.14%), and engraftment syndrome (1/84, 1.19%). In the other 37 patients, diarrhea was due to multiple factors, including infection plus GVHD (13/84, 15.48%), infection plus medication-induced (22/84, 26.19%), infection plus GVHD plus medication-induced (1/84, 1.19%), and GVHD plus medication-induced (1/84, 1.19%) (Fig. 2a). In summary, the primary cause of post-transplantation diarrhea was infection (57/84, 67.86%), followed by medication (38/84, 45.24%) and GVHD (21/84, 25.00%) (Fig. 2b).

To determine whether pre-transplantation colonization with GI pathogens has an impact on post-transplantation diarrhea, we compared the proportion of diarrhea patients in the colonized and non-colonized groups. The incidence of diarrhea was higher in the colonized patients (56/95, 58.95%) than in the non-colonized patients (28/63, 44.44%), although the difference was not significant (*P* = 0.074). However, there was a significant increase in the incidence of infection-induced diarrhea in the colonized group (47/95, 49.47%) compared to the non-colonized group (10/63, 15.87%) (*P* < 0.001, Fig. 2c).

### Pathogens detected by FilmArray GI panel

**Fig. 3:**
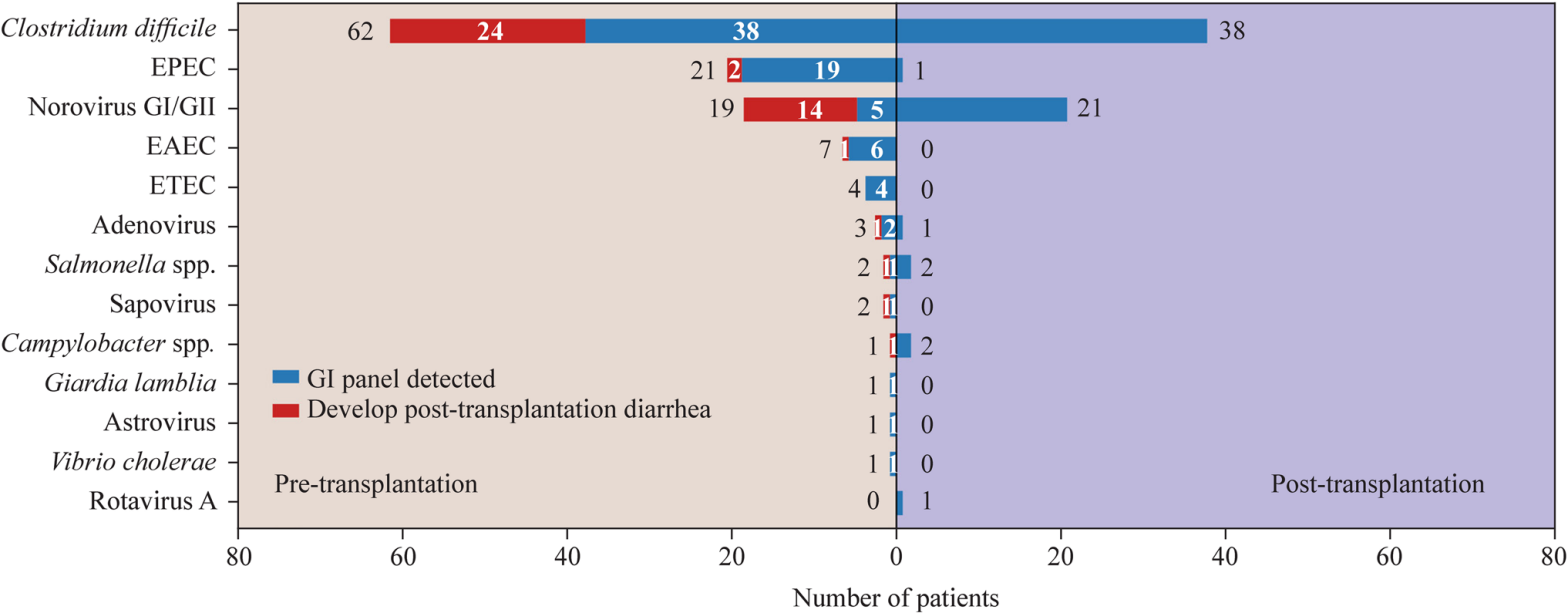
Pathogens detected by FilmArray gastrointestinal (GI) panel in pre- and post-transplantation stool samples. The number of patients who had a clinically relevant post-transplantation diarrheal infection due to pre-transplantation colonization is shown for each pathogen (red part in the bar plot). *EPEC* enteropathogenic *Escherichia coli*, *EAEC* enteroaggregative *E. coli, ETEC* enterotoxigenic *E. coli*

In total, 95 patients were colonized by at least one GI pathogen pre-transplantation, resulting in 124 pathogens being detected. The most commonly detected pathogen was *C. difficile*, present in 62 (65.26%) patients. Other pathogens detected included EPEC (21/95, 22.11%), norovirus (19/95, 20.00%), EAEC (7/95, 7.37%), etc. (Fig. 3, left column). Fifty-nine patients tested positive for at least one GI pathogen 28 ± 7 days after transplantation. The most commonly detected pathogen was *C. difficile*, present in 38 (64.41%) patients, followed by norovirus (21/59, 35.59%) (Fig. 3, right column). Interestingly, we found that EPEC was only present in 1 of the 59 patients. No other pathogenic *E. coli*, including EAEC and EPEC, were detected in the 59 patients. These results indicated that the conditioning therapy as well as other medications used during the transplantation period may eliminate most colonizing bacterial pathogens, with the exception of *C. difficile*. In another way, viral pathogens were almost unaffected.

### Risk of post-transplantation diarrhea due to colonizing pathogens

We next assessed the proportions of patients who developed a diarrheal infection due to their colonized pathogens pre-transplantation. Overall, 45 of the 124 (36.29%) colonized pathogens detected in 39 patients led to post-transplantation diarrhea. In detail, 14 of 19 (73.68%) patients who were initially colonized with norovirus pre-transplantation developed a post-transplantation norovirus infection. Twenty-four of 62 (38.71%) patients colonized with *C. difficile* developed a diarrheal infection. Only 2 of 21 (9.52%) patients colonized with EPEC and 1 of 7 (14.29%) patients colonized with EAEC developed post-transplantation EPEC and EAEC infections, respectively. In addition, four patients were colonized with ETEC, and three patients were colonized with *Giardia lamblia*, astrovirus and *Vibrio cholerae*, but none of them developed post-transplantation diarrhea due to their colonized pathogens (Fig. 3, left column).

**Fig. 4:**
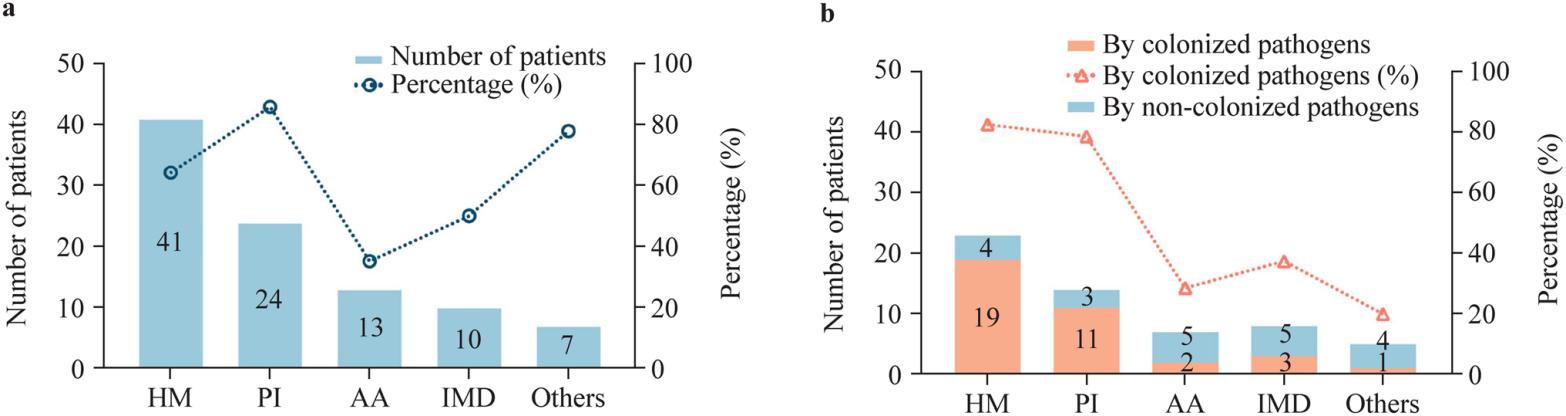
Impact of different underlying diseases on pathogen colonization and subsequent development of infectious diarrhea due to the colonized pathogens. **a** The number and proportions of patients with different underlying diseases who were colonized with gastrointestinal pathogens pre-transplantation; **b** the number and proportions of patients in the colonized group who developed infectious diarrhea due to their colonized pathogens. *HM* hematological malignancies, *PI* primary immunodeficiency, *AA* aplastic anemia, *IMD* inherited metabolic disorders

We also assessed the proportions of patients with different underlying diseases who developed infectious diarrhea due to their colonized pathogens. Twenty-four of 28 (85.71%) patients with primary immunodeficiency were colonized by at least one GI pathogen pre-transplantation. In 14 patients who developed infectious diarrhea post-transplantation, 11 of them (11/14, 78.57%) had colonization by pathogens before transplantation. Similarly, 41 of 64 (64.06%) patients with hematological malignancies were colonized with GI pathogens, and 19 of 23 (82.60%) patients who developed a diarrheal infection were colonized by pathogens (Fig. 4). On the other hand, patients with aplastic anemia and inherited metabolic disorders were much less frequently colonized with GI pathogens (35.14% and 50.00%, respectively, *P* < 0.001), and the risk of developing infectious diarrhea was also comparatively lower (28.57% and 37.50%, respectively, *P* = 0.005) (Fig. 4).

### Comparison of FilmArray GI-detected pathogens with clinic-confirmed pathogens detected during diarrhea episodes

In 57 patients who developed infectious diarrhea posttransplantation, 92 pathogens were detected by various methods and confirmed by clinicians. Norovirus and *C. difficile* were the most frequently detected diarrheal pathogens, present in 26 and 25 patients, respectively. EBV, adenovirus, and *Enterococcus faecium* were present in 7, 5, and 4 patients, respectively. Other pathogenic microbes, such as *Salmonella* spp.*,* CMV, and HHV6, were less frequently detected (Table 2, left part).

**Table 2:** Pathogens detected during diarrhea episodes

Pathogens	Test results				Reason of false results	
	Number of patients, positive/total	Test positive percentage, % (95% CI)	Number of pathogen, positive/total	Pathogen positive percentage, % (95% CI)	Not in GI panel	GI false negative
Norovirus	26/56	46.4 (33.0–60.3)	26/92	28.3 (19.4–38.6)	0	5
*Clostridium difficile*	25/56	44.6 (31.3–58.5)	25/92	27.2 (18.4–37.4)	0	0
EBV	7/56	12.5 (5.2–24.1)	7/92	7.6 (3.1–15.1)	7	0
Adenovirus	5/56	8.9 (3.0–19.6)	5/92	5.4 (1.8–12.2)	0	4
*Enterococcus faecium*	4/56	7.1 (2.0–17.3)	4/92	4.3 (1.2–10.8)	4	0
*Salmonella* spp.	3/56	5.4 (1.1–14.9)	3/92	3.3 (0.7–9.2)	0	0
CMV	3/56	5.4 (1.1–14.9)	3/92	3.3 (0.7–9.2)	3	0
ESBL positive *Escherichia coli*	3/56	5.4 (1.1–14.9)	3/92	3.3 (0.7–9.2)	3	0
HHV6	2/56	3.6 (0.4–12.3)	2/92	2.2 (0.3–7.6)	2	0
EPEC	2/56	3.6 (0.4–12.3)	2/92	2.2 (0.3–7.6)	0	0
*Stenotrophomonas maltophilia*	2/56	3.6 (0.4–12.3)	2/92	2.2 (0.3–7.6)	2	0
Rotavirus	2/56	3.6 (0.4–12.3)	2/92	2.2 (0.3–7.6)	0	2
Human parvovirus B19	2/56	3.6 (0.4–12.3)	2/92	2.2 (0.3–7.6)	2	0
BK virus	1/56	1.8 (0.0–9.6)	1/92	1.1 (0.0–5.9)	1	0
Sapovirus	1/56	1.8 (0.0–9.6)	1/92	1.1 (0.0–5.9)	0	0
*Campylobacter* spp.	1/56	1.8 (0.0–9.6)	1/92	1.1 (0.0–5.9)	0	0
EAEC	1/56	1.8 (0.0–9.6)	1/92	1.1 (0.0–5.9)	0	0
*Weissella confuse*	1/56	1.8 (0.0–9.6)	1/92	1.1 (0.0–5.9)	1	0
*Enterobacter cloacae*	1/56	1.8 (0.0–9.6)	1/92	1.1 (0.0–5.9)	1	0

*GI* gastrointestinal, *EBV* Epstein–Barr virus, *CMV* cytomegalovirus, *ESBL* extended spectrum beta-lactamase*, HHV6* human herpesvirus 6, *EPEC* enteropathogenic *Escherichia coli*, *EAEC* enteroaggregative *E. coli*

## Discussion

In the present study, we evaluated whether the use of the FilmArray GI panel for the management of pediatric HSCT patients can (1) predict post-transplantation infectious diarrhea caused by pre-transplantation colonization and (2) increase the diagnostic yield for patients with diarrhea episodes. It is notable that 60.13% (95/158) of the HSCT patients were colonized with at least one GI pathogen before conditioning therapy, which is higher than the data reported in adult patients [14].

While most of the previous studies focused on *C. difficile* colonization [11, 13, 14], we found in our study that patients colonized by norovirus are at the highest risk of developing diarrhea after transplantation, higher than those colonized by *C. difficile*. Previous studies have shown that hospital transmission of norovirus frequently occurs and causes excess patient morbidity, especially in vulnerable populations [17, 18]. Furthermore, noroviruses are stable in the environment and may be viable for days or longer on various surfaces [19, 20]. Therefore, although norovirus decolonization may be difficult, screening of norovirus before transplantation may have positive implications for infection prevention and control. For patients colonized with norovirus, single room assignment or bedside isolation is recommended to reduce the risk of in-hospital outbreaks [21].

Another dominant colonizing pathogen before transplantation is EPEC, similar to other GI panel studies in immunocompromised patients, which have also reported a surprisingly large percentage of diarrheagenic *E. coli* pathotypes, including EPEC. Such a high detection rate also raised the concern of false positives and possible cross-reactivity because EPEC detection could be due to low-level colonization or false-positive results, possibly owing to the detection of the *eae* gene in other microbes, such as *Aeromonas* spp. [22–24]. Whether there is false-positive EPEC detection in our study awaits further investigation. Nonetheless, the risk of diarrhea caused by EPEC as well as EAEC and ETEC colonization was relatively lower in our study than in Kubiak’s report [14]. We speculated that the conditioning therapy as well as other medications used during the transplantation period may eliminate most bacterial pathogens, including diarrheagenic *E. coli*, thereby reducing the risk of colonization and diarrhea after transplantation.

Studies focusing on post-transplantation diarrhea in adult patients have suggested that non-infectious causes predominated, and fewer patients had an infectious cause for their diarrhea (13%–40%) [1, 25]. Interestingly, recent studies have reported that the cause of GI disease remains undefined in 60%–83% of solid organ transplant recipients [26, 27]. The low detection rate of traditional diagnostic methods, which rely on culture, microscopy or antigen detection techniques, may be one of the reasons for these contradictory conclusions. With the rapid development of new diagnostic technologies (including RT‒qPCR, multiplexed PCR, and mNGS), our understanding of the etiologies of diarrhea has improved [9, 28]. In this study, we comprehensively analyzed the etiologies of post-transplantation diarrhea in pediatric HSCT patients and found that only 50.00% of patients developed diarrhea due to a single factor. For the other 50.00% of patients, diarrheal symptoms were multifactorial, and positive results from microbiological tests did not rule out other clinically important causes (GVHD, medication-induced diarrhea, etc.). This observation is in accordance with recent data showing that the use of multiplexed PCR panels improves the diagnostic accuracy of infectious diarrhea, thus facilitating accurate and effective clinical therapeutic decision-making [4]. However, outcome studies demonstrating the overall clinical benefit of using the GI panel for transplant patients are still lacking and much needed.

Although FilmArray GI panel testing improves the diagnostic yield by almost twofold in our study (55/92, 59.78% vs. 30/92, 32.61%), the current version of the FilmArray GI panel cannot fully meet the pathogen detection needs and may decrease its utility in HSCT patients. For example, CMV and EBV are common causes of infectious diarrhea in HSCT patients, but most U.S. Food and Drug Administration-cleared GI panels (including the FilmArray GI panel) do not target those pathogens. In our study, nine patients developed infectious diarrhea due to CMV or EBV infection, and all of these pathogens were detected by either RT‒qPCR or mNGS. In addition, although adenovirus is included in the FilmArray GI panel menu, detection is limited to two specific serotypes, F40/F41, which represent a minority of clinically relevant adenovirus (ADV) species in HSCT patients [29, 30]. McMillen et al. reported that in cancer patients with ADV infections, ADV species C accounted for 54% of the species compared to only 14% for ADV species F in stool samples [30]. The limited ability to detect ADV in pediatric HSCT patients was also evident in our study, where 4 (80.00%) cases of proven ADV infections were missed by the FilmArray GI panel. For patients at high risk of ADV infections, it is necessary to use other tests, such as antigen detection or RT‒qPCR, to be able to detect all clinically relevant ADV species.

The underlying disease type of HSCT patients can also affect the cause of post-transplantation diarrhea [31, 32]. Compared to patients with aplastic anemia and inherited metabolic disorders, patients with primary immunodeficiency and hematological malignancies have a much higher risk of pre-transplantation pathogen colonization, leading to a higher proportion of post-transplantation infectious diarrhea. As both primary immunodeficiency and hematological malignancy patients were immunocompromised and may have a complex hospitalization history, screening of colonized pathogens in these patients may lead to increased clinical benefits and optimized healthcare resource utilization.

In summary, our data show that more than half of pediatric patients who were admitted for HSCT were colonized with various gastrointestinal pathogens, and more than one-third of these pathogens were associated with posttransplantation diarrhea. In addition, the FilmArray GI panel can increase the detection rate of diarrheal pathogens in pediatric HSCT patients, but the panel needs to be optimized for pathogen species, and further studies assessing its clinical impact and cost-effectiveness in this specific patient population are also needed.

## Data Availability

The data that support the findings of this study are not openly available due to reasons of sensitivity and are available from the corresponding author upon reasonable request. Data are located in controlled access data storage at Shanghai Children’s Medical Center.
